# Three-Dimensional Printed Cell-Adaptable Nanocolloidal Hydrogel Induces Endogenous Osteogenesis for Bone Repair

**DOI:** 10.34133/bmr.0146

**Published:** 2025-02-14

**Authors:** Wenxin Lu, Li Li, Ruyi Wang, Yanting Wu, Yao Chen, Bowen Tan, Zhihe Zhao, Maling Gou, Yu Li

**Affiliations:** ^1^ State Key Laboratory of Oral Diseases & National Center for Stomatology & National Clinical Research Center for Oral Diseases & Department of Orthodontics, West China Hospital of Stomatology, Sichuan University, Chengdu 610041, Sichuan, China.; ^2^ Sichuan Hospital of Stomatology, Chengdu 610015, Sichuan, China.; ^3^ Department of Biotherapy, Cancer Center and State Key Laboratory of Biotherapy, West China Hospital, Sichuan University, Chengdu 610041, Sichuan, China.

## Abstract

Repairing critical bone defects remains a formidable challenge in regenerative medicine. Scaffolds that can fill defects and facilitate bone regeneration have garnered considerable attention. However, scaffolds struggle to provide an ideal microenvironment for cell growth and differentiation at the interior of the bone defect sites. The scaffold’s structure must meet specific requirements to support endogenous bone regeneration. Here, we introduce a novel 3D-printed nanocolloidal gelatin methacryloyl (GelMA) hydrogel, namely, the nG hydrogel, that was derived from the self-assembly of GelMA in the presence of Pluronics F68, emphasizing its osteoinductive capability conferred solely by the specific nanocolloidal structure. The nG hydrogel, exhibiting remarkable pore connectivity and cell-adaptable microscopic structure, induced the infiltration and migration of rat bone mesenchymal stem cells (rBMSCs) into the hydrogel with a large spreading area in vitro. Moreover, the nG hydrogel with interconnected nanospheres promoted the osteogenic differentiation of rBMSCs, leading to up-regulated expression of ALP, RUNX2, COL-1, and OCN, as well as augmented formation of calcium nodules. In the critical-sized rat calvarial defect model, the nG hydrogel demonstrated improved repair of bone defects, with enhanced recruitment of endogenous CD29^+^ and CD90^+^ stem cells and increased bone regeneration, as indicated by significantly higher bone mineral density (BMD) in vivo. Mechanistically, the integrin β1/focal adhesion kinase (FAK) mechanotransduction signaling pathway was up-regulated in the nG hydrogel group both in vitro and in vivo, which may partially account for its pronounced osteoinductive capability. In conclusion, the cell-adaptable nG hydrogel shows great potential as a near-future clinical translational strategy for the customized repair of critical-sized bone defects.

## Introduction

Regeneration of critical or large-sized bone defects remains a great challenge. While autogenous bone grafting is considered the gold standard, it has limitations such as short supply and causing secondary trauma to patients [1,2]. Additionally, the shape of obtained autogenous bone often fails to accurately match the defect site, further restricting its clinical application [3]. Scaffolds in bone tissue engineering have emerged as a promising solution, as they can well fill bone defect sites. However, these scaffolds still face challenges in providing ideal microenvironments for cell growth and bone regeneration, particularly within the interior of defect sites. Barriers within scaffolds can restrict cell motility, including spreading and migration, and cells are more prone to death due to poor supply of nutrients and oxygen. Consequently, cells struggle to grow and differentiate at the center of bone defects, impairing their ability to function and ultimately form new bone [4,5].

Guiding endogenous stem cells to infiltrate into the scaffold during the bone repair process, followed by robust proliferation, migration, and osteogenic differentiation within the scaffold, enables more efficient and functional repair of critical or large-sized bone defects. To achieve this functionality, the scaffold’s structure must meet specific requirements [6]. It is well known that a porous structure facilitates cell proliferation, migration, spreading, differentiation, and nutrient exchange, expediting bone regeneration. However, while conventional porous scaffolds provide space for cells, the walls between pores still hinder various cell functions [7]. Therefore, the porous structure with enhanced connectivity is required to allow unobstructed infiltration of cells. Moreover, while a porous structure offers passive channels for cell motility, the ability to adapt to cellular activities is an intrinsic characteristic of the native extracellular matrix (ECM). Therefore, a dynamic microscopic structure that provides an adaptive microenvironment for cell spreading and migration could enhance cell–cell and cell–ECM interactions, thereby promoting the biofunctions of stem cells [8,9].

In addition to stem cell infiltration and migration, osteogenic differentiation is essential for bone regeneration. Currently, various bioactive factors are incorporated into scaffolds to enhance bone repair. However, exogenous cytokines used to recruit stem cells and direct cell differentiation, such as SDF-1, vascular endothelial growth factor (VEGF), and bone morphogenetic protein-2 (BMP-2), face challenges due to high costs and potential risks of tissue overgrowth [10]. Nanostructures, known for their ability to increase nano-roughness related to biomechanics and provide more adhesion sites for both organic and inorganic elements with long-term effectiveness, have been found to strongly influence cell behaviors, particularly the differentiation fate of stem cells [11]. Scaffolds with nanostructures that can significantly enhance cell–material interactions at the nanoscale have been extensively utilized in bone regeneration [12,13].

**Fig. 1.**
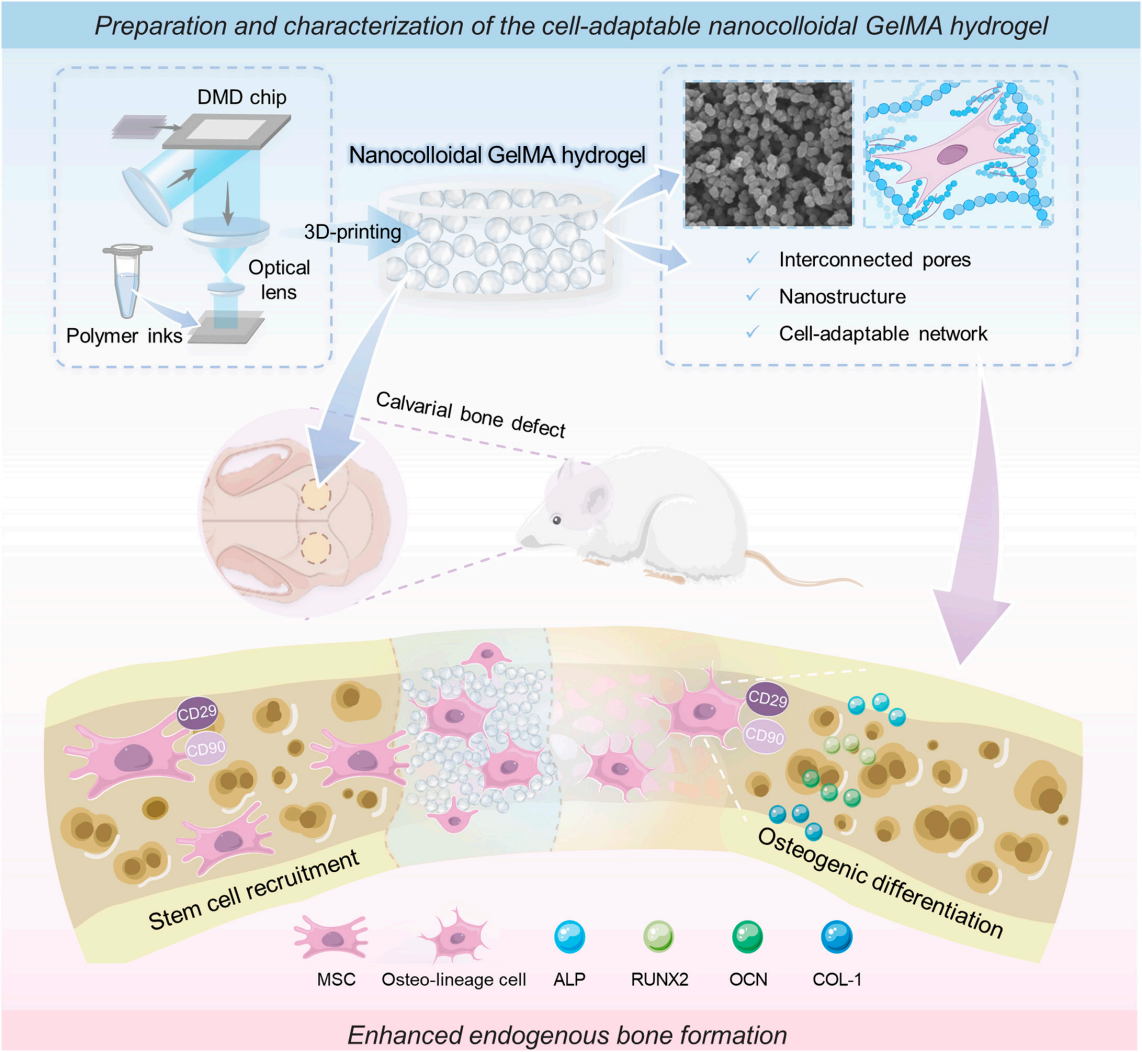
The conceptual schematic illustrates the potential applications of the cell-adaptable nG hydrogel in bone regeneration. Serving as an osteoinductive bio-sponge, the nG hydrogel adeptly recruits endogenous stem cells to the defect area. Subsequently, it fosters their viability, migration, and osteogenic differentiation, synergistically enhancing the overall process of bone regeneration.

In this study, we introduced a 3-dimensional (3D) printed nanocolloidal gelatin methacryloyl (GelMA) hydrogel (the nG hydrogel) obtained from the self-assembly and photo-crosslinking of GelMA nanospheres in the presence of Pluronics F68 (F68). The nanocolloidal structure of the nG hydrogel exhibited specific properties. First, the nanocolloidal structure endowed the nG hydrogel with interconnected pores and cell-adaptable capability. The cell-adaptable porous structure could provide dynamic spaces that adapted to cell behaviors, enabling the infiltration of stem cells into the hydrogel’s interior and enhancing its effectiveness in repairing critical or large-sized bone defects. Second, the nanostructure of the nG hydrogel promoted osteogenic differentiation of stem cells through biomechanical stimulation. The cell-adaptable structure further enhanced cellular perception of the ECM and intercellular communication, thereby fostering its osteoinductive ability. In rat critical-sized bone defect models, the 3D-printed nG hydrogel acted as a customized osteoinductive sponge, effectively enhancing bone repair by facilitating the recruitment and osteogenic differentiation of endogenous stem cells without the need for additional bioactive factors (Fig. 1). Thus, the 3D-printed nG hydrogel exhibits a unique nanostructure that enhances key biofunctions of mesenchymal stem cells, including migration, spreading, and osteoblastic differentiation, due to its interconnectivity, roughness, and cell-adaptive capacity. This suggests promising potential for the clinical translation of 3D-printed nanocolloidal hydrogels in bone regeneration.

## Materials and Methods

### 3D printing procedure

The 3D printing experiments in this study were conducted using a self-built digital light projection (DLP)-based printer, as previously reported [14,15]. In detail, the DLP-based printer comprises a 405-nm visible-light-emitting diode with an intensity of 130 mW/cm^2^, along with a digital micromirror device (DMD) chip with a resolution of 50 μm. GelMA with the degree of methacrylation of 97% and photo-initiator lithium phenyl-2,4,6-trimethyl-benzoylphosphinate (LAP) were synthesized following previously established protocols [16]. Polymer inks for 3D printing were prepared as follows: Initially, lyophilized GelMA was dissolved in phosphate-buffered saline (PBS) containing 0.75% w/v LAP at a concentration of 15% w/v. F68 (Meilunbio, China) and polyethylene oxide (PEO; Macklin, China) powders were separately dissolved in PBS to achieve final concentrations of 15% w/v and 1.5% w/v, respectively.

For the 3D printing of GelMA (G), porous GelMA (pG), and nG hydrogels, a 15% w/v GelMA solution containing 0.75% w/v LAP was separately mixed with PBS, PEO, and F68 solutions in a 2:1 volume ratio to obtain the respective polymer inks. After ensuring uniform mixing, the polymer ink was deposited onto a hydrophobic glass slide for DLP-based 3D printing. The 3D printing process was initiated by inputting images into an operating computer, which then projected the patterned light onto the surface. The thickness of printed constructs was controlled by adjusting the spacing between 2 glass plates at both ends of the constructs. Upon 10 to 30 s of exposure to the patterned light, the ink underwent selective photo-crosslinking, forming the pre-designed shapes. Afterward, the 3D-printed hydrogels were washed in PBS 3 times, each for 10 min.

For 3D printing involving living cells, the procedure was conducted under aseptic conditions. All solutions were sterilized using 0.22-μm syringe filters (Millipore, USA) before mixing to generate the polymer ink. The bioink was prepared by resuspending cells in the polymer ink at a density of 5 × 10^6^/ml. The 3D-printed cellular constructs were then washed in PBS 3 times, each for 10 min at 37 °C. After that, the constructs were cultured in a medium at 37 °C with 5% CO_2_. The next day, the culture medium was replaced to completely remove PEO, F68, and other unreacted molecules.

### Characterization of 3D-printed hydrogels

Bright-field images of 3D-printed hydrogels with predefined shapes were captured using super-resolution structured illumination microscopy (SIM; Nikon, Japan). The field emission scanning electron microscope (FE-SEM; Thermo Fisher Scientific, USA) characterized the morphology of the 3D-printed constructs at an operating voltage of 15 kV. Samples were fractured in liquid nitrogen and then dehydrated with graded alcohols, supercritical dried, and sputter-coated with gold. For FE-SEM imaging of the cell-laden constructs, samples were fixed with 4% paraformaldehyde before cryogenic fracturing. The nanoscale geometry was assessed using atomic force microscopy (AFM; SPM9600, Shimadzu, Japan). Image analysis was conducted using ImageJ software [National Institutes of Health (NIH), USA].

To examine bioactivity, samples were immersed in 1.5× simulated body fluid (SBF) at 37 °C. After 24 h, the samples were removed, washed with distilled water, and dried. The deposited minerals were then characterized using a FE-SEM coupled with an energy-dispersive spectrometer (FE-SEM/EDS; Oxford INCA, UK).

The mechanical properties of hydrogels were evaluated using a rheometer (Anton Paar MCR 302e, Austria). For the tests, 3D-printed cylindrical hydrogels (Φ25 mm × 0.6 mm) were placed on a parallel plate with a 25-mm diameter in oscillatory mode. Initially, amplitude sweeps (10 rad/s, 0.01% to 100% strain) were conducted to determine the linear viscoelastic regime of the samples. Subsequently, a constant strain (0.1% strain) within this range was selected for frequency sweeps (100 to 0.1 rad/s). This methodology was employed to accurately measure the storage (*G*′) and loss (*G*″) moduli of the hydrogels.

Cell viability was assessed at 1, 4, and 7 d of incubation using live/dead staining (Thermo Fisher Scientific, USA) and the Cell Counting Kit-8 proliferation assay (CCK-8; Dojindo Molecular Technologies, Japan), following the manufacturer’s protocols.

### Cell migration and spreading

Primary rat bone mesenchymal stem cells (rBMSCs) were harvested from the tibia region of 2-week-old male Sprague–Dawley (SD) rats. The capabilities for cell spreading and migration were assessed both on the surface of and within the hydrogels. For cells cultured on hydrogels, hydrogels with a vertical height of 200 μm were engineered on adhesive glass substrates using cell-free bioink. Cell suspensions with a concentration of 5 × 10^4^/ml were then seeded onto the hydrogels in 24-well plates. For cells cultured within hydrogels, cellular constructs also measuring 200 μm in height were prepared on adhesive glass substrates using the bioink described in the “3D printing procedure” section. After 3 d, cellular morphology and spatial distribution were evaluated using FE-SEM and cytoskeletal fluorescence staining. For cytoskeletal fluorescence staining, cells were initially fixed with 4% paraformaldehyde. They were then blocked with 3% bovine serum albumin (BSA) solutions for 1 h before being incubated with Phalloidin-iFluor488 (1:800, Solarbio, China) for an additional hour. Images were captured using laser scanning confocal microscopy (LSCM; OLYMPUS, Japan).

The microscopic structural dynamics of the nG hydrogel was recorded using a super-resolution SIM (Nikon, Japan). For visualization, a fluorescein isothiocyanate (FITC)-labeled GelMA solution (15% w/v, containing 0.75% LAP and 0.3% w/v FITC-GelMA) was used for 3D printing with cells. Before 3D printing, this FITC-labeled polymer ink was used to resuspend CT26 cells, which were labeled with fluorescent red proteins, at a concentration of 1 × 10^7^/ml. The bioink containing cells was then thoroughly mixed and added to a confocal dish, followed by a 15 s exposure to patterned light. After washing, the resultant FITC-labeled nG hydrogels containing cells marked with fluorescent red proteins were cultured in medium. Imaging of the cells and hydrogels was performed after 2 d of culture. To monitor the dynamic motility of cells and changes in the nanostructure of the nG hydrogel, time-lapse images were captured using SIM at 40-min intervals for at least 4 h.

### Osteogenic activity

To investigate the osteogenic differentiation of rBMSCs in hydrogels, an osteogenic induction medium was employed. This medium comprised 100 nM dexamethasone (Sigma-Aldrich, USA), 10 mM β-glycerophosphate (Sigma-Aldrich, USA), and 50 μg/ml ascorbic acid (Sigma-Aldrich, USA). The medium was refreshed every 2 d. On days 7 and 14, alkaline phosphatase (ALP) staining and quantitative analysis were conducted using the BCIP/NBT kit (Beyotime, China) and the ALP assay kit (Beyotime, China), respectively. The intracellular total protein content was calibrated using the bicinchoninic acid (BCA) protein assay kit (Beyotime, China). On day 21, alizarin red staining (ARS; Sigma-Aldrich, USA) was employed to assess the formation of mineralization nodules. The stained samples were captured using an inverted optical microscope (OLYMPUS IX71, Japan). Following ARS, 10% w/v cetylpyridine chloride (CPC) solutions (Sigma-Aldrich, USA) were introduced for a 30-min shock, and quantitative analysis was subsequently performed using a stereomicroscope (Thermo Fisher Scientific, USA).

To evaluate the expressions of osteogenic proteins, runt-related transcription factor 2 (RUNX2) and osteocalcin (OCN), immunocytochemistry (ICC) staining was performed. After 14 d of osteogenic induction, fixed samples were permeabilized with 0.1% Triton X-100, blocked with 3% BSA solution, and then incubated overnight at 4 °C with primary antibodies against RUNX2 (1:200, Huaanbio, China) and OCN (1:100, Huaanbio, China). Subsequently, the samples were incubated with Alexa Fluor 594 secondary antibodies (1:250, Huaanbio, China), followed by nuclear labeling with 4′,6-diamidino-2-phenylindole (DAPI) solution (10 μg/ml, Solarbio, China) for 5 min. After washing, the samples were immersed in PBS and visualized using a confocal laser microscope (CLSM; LSM880, Zeiss, Germany). The captured images were analyzed using ImageJ software (NIH, USA).

Additionally, the gene expression of *Runx2*, *Alpl*, *Bglap*, and *Col1a1* was assessed using quantitative real-time polymerase chain reaction (qRT-PCR) after 14 d of osteogenic induction. For this analysis, total RNA was extracted using TRIzol reagents (Invitrogen, USA). The gene expression analysis was conducted using the ABI PRISM 7300 sequence detection system (Applied Biosystems, Thermo Fisher Scientific, USA). The process utilized the PrimeScript RT Reagent Kit with gDNA Eraser (Perfect Real Time, TaKaRa, Japan) and TB Green Premix Ex Taq II (Tli RNaseH Plus, TaKaRa, Japan). The relative expression levels of target genes were calculated using the 2^ΔΔCt^ method. Detailed primer sequences were provided in Table S1.

### Expression of mechanotransduction-related factors

To further assess the interaction between cells and hydrogels, gene expression of *itgb1* and *Ptk2* was analyzed using qRT-PCR, while protein expression of integrin β1 and phosphorylated focal adhesion kinase (pFAK) was examined using Western blot (WB) and ICC staining, respectively. For qRT-PCR, the protocol was identical to the one described previously. Detailed primer sequences for this analysis are provided in Table S1. For WB analysis, after total protein extraction, primary antibodies were used as follows: integrin β1 (1:2,000, Huaanbio, China), FAK (1:1,000, Huaanbio, China), pFAK (1:1,000, Huaanbio, China), and β-actin (1:5,000, Proteintech, USA). Horseradish peroxidase (HRP)-conjugated goat anti-rabbit antibody (1:5,000, Huaanbio, China) and HRP-conjugated goat anti-mouse antibody (1:5,000, Huaanbio, China) were used as secondary antibodies. The WB bands were visualized using the ChemiDoc Touch Imaging System (Bio-Rad, USA). For ICC staining, samples were processed according to standard protocols previously described. Specifically, cells were incubated overnight at 4 °C with primary antibodies against integrin β1 (1:50) and pFAK (1:50). Captured images were subjected to image processing and analysis using ImageJ software (NIH, USA).

### Animal study

All animal experiments complied with the Animal Research: Reporting of In Vivo Experiments (ARRIVE) guidelines. The Animal Care and Ethics Committee of West China School of Stomatology, Sichuan University, approved all animal study protocols (WCHSIRB-D-2022-583).

To evaluate the in vivo degradability and biocompatibility, 100 μl of hydrogels, each with a diameter of 8 mm and a height of 2 mm (Fig. S1), was implanted in the dorsal region of 8-week-old male Balb/c mice (weight range: 20 to 25 g). Skin specimens were collected at 7, 14, 28, and 56 d to observe the degradation of hydrogels and subcutaneous tissue inflammation. These samples were documented through photography and subsequently fixed in 10% neutral formalin (Solarbio, China) for histological examination using hematoxylin and eosin (H&E) staining.

For the evaluation of bone regeneration, 8-week-old male SD rats (weight range: 200 to 220 g) were utilized. Under isoflurane anesthesia (RWD, China), full-layer calvarial bone defects were created on both sides of the middle suture using a bone drill with an external diameter of 5 mm. The rats were divided into 4 groups based on the implantation material: the blank control, G hydrogel, pG hydrogel, and nG hydrogel. Each implanted hydrogel was designed to be 5 mm in diameter and 0.8 mm in height.

At 1, 4, and 10 weeks after surgery, skull samples were collected and fixed in 10% neutral formalin. Bone formation was evaluated using micro-computed tomography (μCT) (μCT80, Scanco Medical, Switzerland) at a high-resolution setting (10-μm voxel size). 3D reconstructed images were analyzed using image analysis software (Scano Medical Evaluation & Visualizer, Scano Medical, Switzerland). Parameters such as bone volume to total tissue volume ratio (BV/TV) and bone mineral density (BMD) were computed.

After scanning, the samples underwent a 2-month decalcification period in 10% EDTA, followed by staining with H&E and Masson’s trichrome (Solarbio, China). Immunohistochemical (IHC) staining involved overnight incubation of tissue sections at 4 °C with primary antibodies against CD29 (1:200, Huaanbio, China), CD90 (1:200, Huaanbio, China), OCN (1:250, Huaanbio, China), COL-1 (1:500, Huaanbio, China), pFAK (1:200, Huaanbio, China), and SDF-1α (1:100, Invitrogen, USA), respectively. This was followed by incubation with the appropriate secondary antibody (ZSGB-BIO, China). The slices were observed using a scanner (Panoramic MIDI, 3DHISTECH, Hungary), and the staining intensity was quantified using ImageJ software (NIH, USA).

### Statistical analysis

Data are presented as the mean ± SD. Statistical analysis was conducted using SPSS 22.0 (IBM, USA). One-way analysis of variance followed by the Student–Newman–Keuls method was used to determine hydrogel differences. Statistical significance was set at *P* < 0.05.

## Results

### Preparation and characterization of the nG hydrogel

**Fig. 2.**
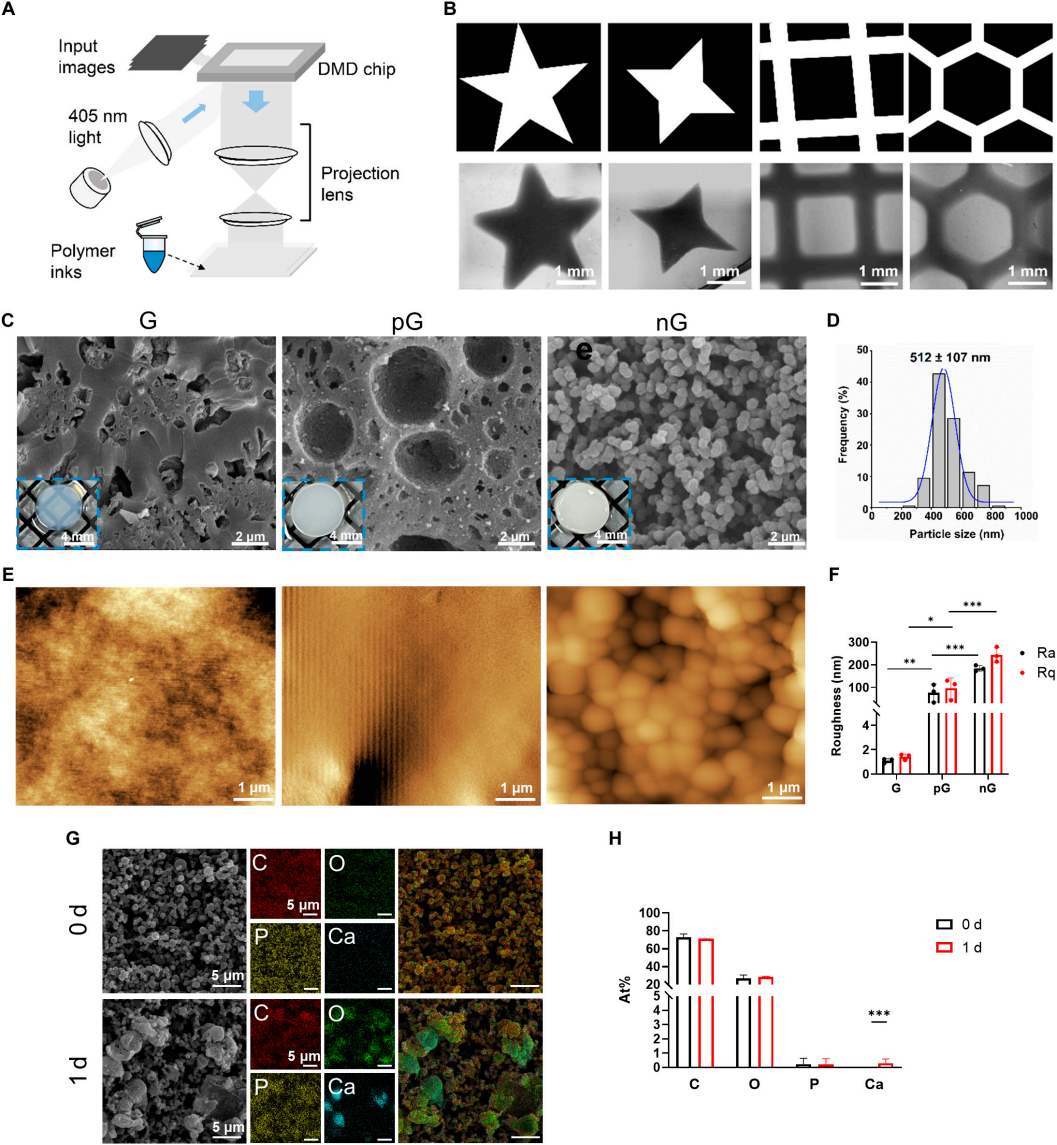
Preparation and characterization of hydrogels. (A) Schematic diagram of DLP-based 3D printing. (B) Personalized 3D printing capability of the nG hydrogel. Upper line, input images for 3D printing; lower line, optical micrographs of the 3D-printed nG hydrogel. (C) Photographs and SEM images showcasing the morphology and microstructures of the hydrogels. (D) Size of nanospheres in the nG hydrogel. (E) AFM images of hydrogels. (F) Ra and Rq obtained from AFM analysis (*n* = 3). (G) EDS elemental mapping images of the nG hydrogel before and after being mineralized in 1.5× SBF for 1 d. (H) Atom percentages of carbon (C), oxygen (O), phosphorus (P), and calcium (Ca) in the nG hydrogel, according to EDS analysis. Statistical significance at *P < 0.05, **P < 0.01, and ***P < 0.005.

To fabricate the nG hydrogel, a polymer ink consisting of 15% w/v GelMA and 15% w/v F68 solution in a 2:1volume ratio was used for DLP-based 3D printing (Fig. 2A). To characterize the microstructure of the nG hydrogel, DLP 3D-printed conventional G hydrogel and pG hydrogel, derived from the formulation of a mixture of 15% w/v GelMA and PBS, and 15% w/v GelMA and 1.5% w/v PEO with the volume ratio of 2:1, respectively, were used as controls.

Images of various shapes were employed in the DLP-based 3D printing process to evaluate the potential of the nG hydrogel to be moldable to complex shapes. Following printing, the hydrogels exhibited geometries consistent with the designs (Fig. 2B). These data suggested that the DLP-based 3D printing technology allowed the manufacture of personalized nG hydrogel that matched the patient’s injury sites. The obtained nG hydrogel featured a white and nontransparent appearance (Fig. 2C), indicating light scattering and the presence of intricate internal architecture. SEM images revealed that the nG hydrogel exhibited a nanocolloidal structure with interconnected nanospheres and pores. In contrast, the G hydrogel showed a dense structure without obvious pores, while the pG hydrogel presented a honeycomb-like porous architecture (Fig. 2C).

The formation mechanism of nanocolloidal structure of the nG hydrogel was driven by polymer immiscibility between GelMA and F68 in aqueous solutions. Specifically, GelMA-rich nanodroplets formed within the F68-rich phase, which then gelled together through self-assembly and photo-crosslinking, facilitated by hydrophobic interaction of photo-crosslinkable methacrylation groups. After removing F68 by leaching with PBS solution, these gelled GelMA nanospheres formed the nanocolloidal networks of the nG hydrogel [15,17]. Similarly, the pG hydrogel was formed through immiscibility between GelMA and PEO in aqueous solutions [18,19]. The GelMA phase was crosslinked into polymeric networks, while the discontinuous PEO droplets were removed by leaching, resulting in a honeycomb-like porous microstructure due to the enhanced hydrogen bonding between gelatin chains in the presence of PEO [19]. In contrast, the formation of the G hydrogel does not involve immiscibility between GelMA and PBS, leading to a relatively dense microstructure of covalently crosslinked GelMA polymeric networks.

Beyond the porous architecture, the nanocolloidal structure of the nG hydrogel composed of interconnected nanospheres (512 ± 107 nm in diameter) (Fig. 2D) increased the specific surface area and roughness (1.1 ± 0.2 nm, 75.6 ± 39.7 nm, and 184.1 ± 12.8 nm for Ra and 1.4 ± 0.2 nm, 95.3 ± 46.5 nm, and 243.9 ± 33.0 nm for Rq of the G, pG, and nG hydrogel, respectively) (Fig. 2E and F). This structure enables the nG hydrogel to adsorb more Ca^2+^ after immersion in the SBF (Fig. 2G). The Ca/P ratio in the nG hydrogel increased from 0 to 0.31 ± 0.54 after 1 d, while in the G and pG hydrogels, it was 0.09 ± 0.15 and 0, respectively (Fig. 2H and Fig. S2).

Frequency sweep analysis (0.1% strain) revealed that the *G*′ of the nG hydrogel was below 1,000 Pa within the frequency range of 0.1 to 100 rad/s. Moreover, the storage (*G*′) and loss (*G*″) moduli of the nG hydrogel exhibited a clear frequency dependence (with a frequency of >15.8 rad/s) compared to the G and pG hydrogels, indicating the dynamic nature of its microstructure (Fig. S3).

The 3D-printed nG hydrogel, with its specific nanocolloidal structure composed of interconnected nanospheres, exhibited an interconnected porous structure and dynamic microscopic structure, showing promise in personalized bone defect repair.

### Biocompatibility of the nG hydrogel

**Fig. 3.**
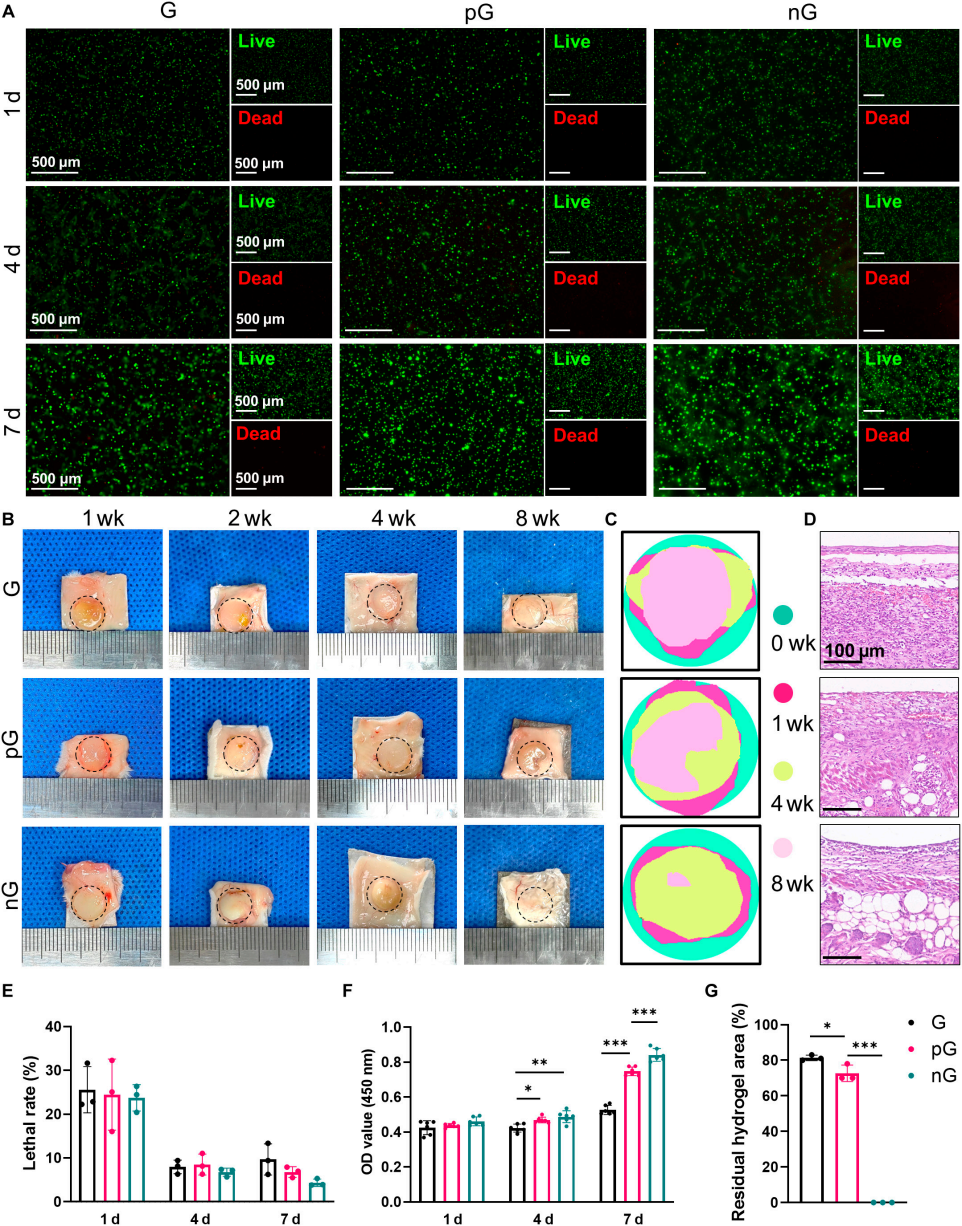
Biocompatibility and biodegradability of the nG hydrogel. (A) Live/dead cell staining (*n* = 3). (B) Degradation behavior of hydrogels in vivo. Black circles indicate the original size of hydrogels. (C) Residual hydrogel traces in the degradation process according to Fig. 2B. (D) H&E staining of subcutaneous tissue around hydrogels after 1-week implantation. (E) Quantitative analysis of live/dead cell staining (*n* = 3). (F) Cell proliferation ability as determined by the CCK-8 assay (*n* = 6). (G) Quantification of the relative residual hydrogel area after 8-week implantation (*n* = 3). Statistical significance at *P < 0.05, **P < 0.01, and ***P < 0.005.

To characterize the biocompatibility of the nG hydrogel in vitro, rBMSCs were cultured within hydrogels using 3D bioprinting techniques. The nG hydrogel not only demonstrated superior cell viability but also significantly enhanced cell proliferation compared to the G and pG hydrogels. This was evidenced by infrequent incidents of cell death observed at 1, 4, and 7 d (Fig. 3A and E) and the results of the CCK-8 assay, which showed a 2.1-, 2.6-, and 3.3-fold increase for the G, pG, and nG hydrogel by day 7, respectively (Fig. 3F). In terms of in vivo biocompatibility, 3D-printed hydrogels without cells were implanted subcutaneously. There was no noticeable inflammation in the surrounding tissues (Figs. 2D and 3B) or vital organs after 1 week (Fig. S4). Moreover, the nG hydrogel exhibited a judicious degradation process conducive to bone regeneration, as partially degrading at 28 d and completely at 56 d (Fig. 3B, C, and G).

### The cell-adaptable nG hydrogel promoted migration and spreading of rBMSCs

**Fig. 4.**
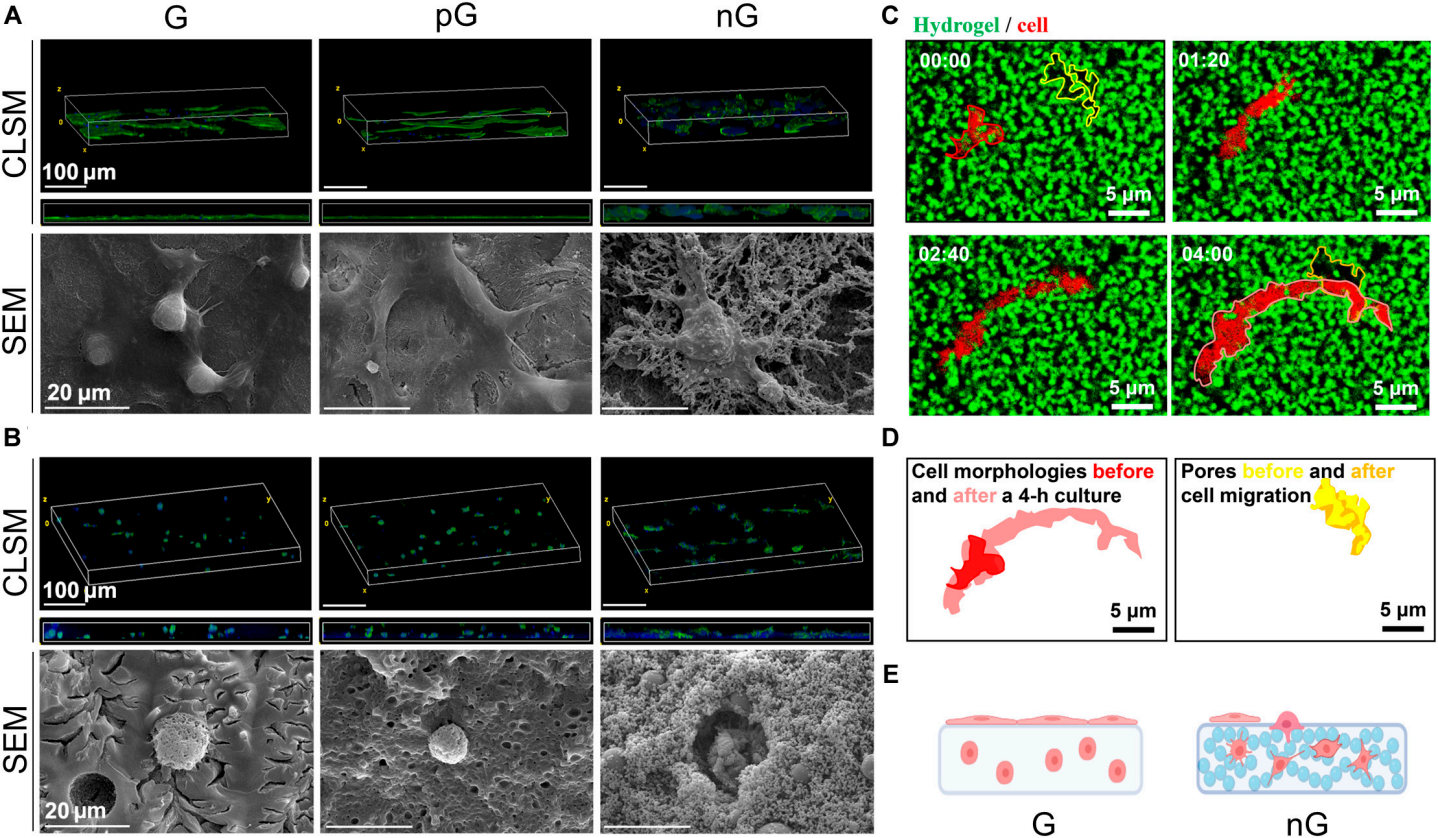
The cell-adaptable nG hydrogel promoted cell adhesion, spreading, and migration. (A) Adhesion, spreading, and migration of rBMSCs on the hydrogels after 3 d culture. Upper line, 3D and *XY* view of CLSM images; lower line, SEM images. (B) Spreading and migration of rBMSCs within the hydrogels after 3 d culture. Upper line, 3D and *XY* view of CLSM images; lower line, SEM images. (C) Time-lapse images of cells in the nG hydrogel captured by SIM. Times are indicated in hour:min. (D) Schematic illustrations of dynamic changes of cells and the nG hydrogels according to (C). (E) Schematic representation of cell adhesion, spreading, and migration on and within hydrogels (created with BioRender.com).

In this study, we initially seeded rBMSCs onto 3D-printed hydrogels. SEM images and fluorescent staining of the cell cytoskeleton revealed distinct cell patterns on the G, pG, and nG hydrogels. Within the first 3 h, SEM images indicated a higher number of cells on the surface of the nG hydrogel. Additionally, while rBMSCs on the G and pG hydrogels spread across the surface, those on the nG hydrogel showed fusion with the hydrogel surface (Fig. S5). After 3 d, rBMSCs on the G and pG hydrogels continued to adhere to their smooth surfaces, resembling 2D cell cultures. In contrast, cells residing on the nG hydrogel exhibited a marked propensity to migrate toward its interior (Fig. 4A). Subsequently, rBMSCs were cultured within the hydrogel using 3D bioprinting techniques. In the G and pG hydrogels, rBMSCs maintained a spherical morphology, contrasting sharply with the expansive and dendritic spreading observed in the nG hydrogel (Fig. 4B).

Moreover, considering the dynamic microscopic structure of the nG hydrogel confirmed by rheological tests and unique cell behaviors observed within different hydrogels, we further explored the cell-adaptable capability of the nG hydrogel. This was achieved using FITC-labeled nG hydrogel (in green) and fluorescent protein-expressing cells (in red) via 3D bioprinting. Time-lapse SIM images captured over a 4-h period showed dynamic changes in both cell morphologies and hydrogel microstructures. In the nG hydrogel, cells migrated and altered their morphology freely, with pores expanding and merging (Fig. 4C and D). These observations indicate that the nG hydrogel possessed a dynamic nanocolloidal structure well-suited to various cellular behaviors (Fig. 4E).

### The cell-adaptable nG hydrogel enhanced osteogenic differentiation of rBMSCs

**Fig. 5.**
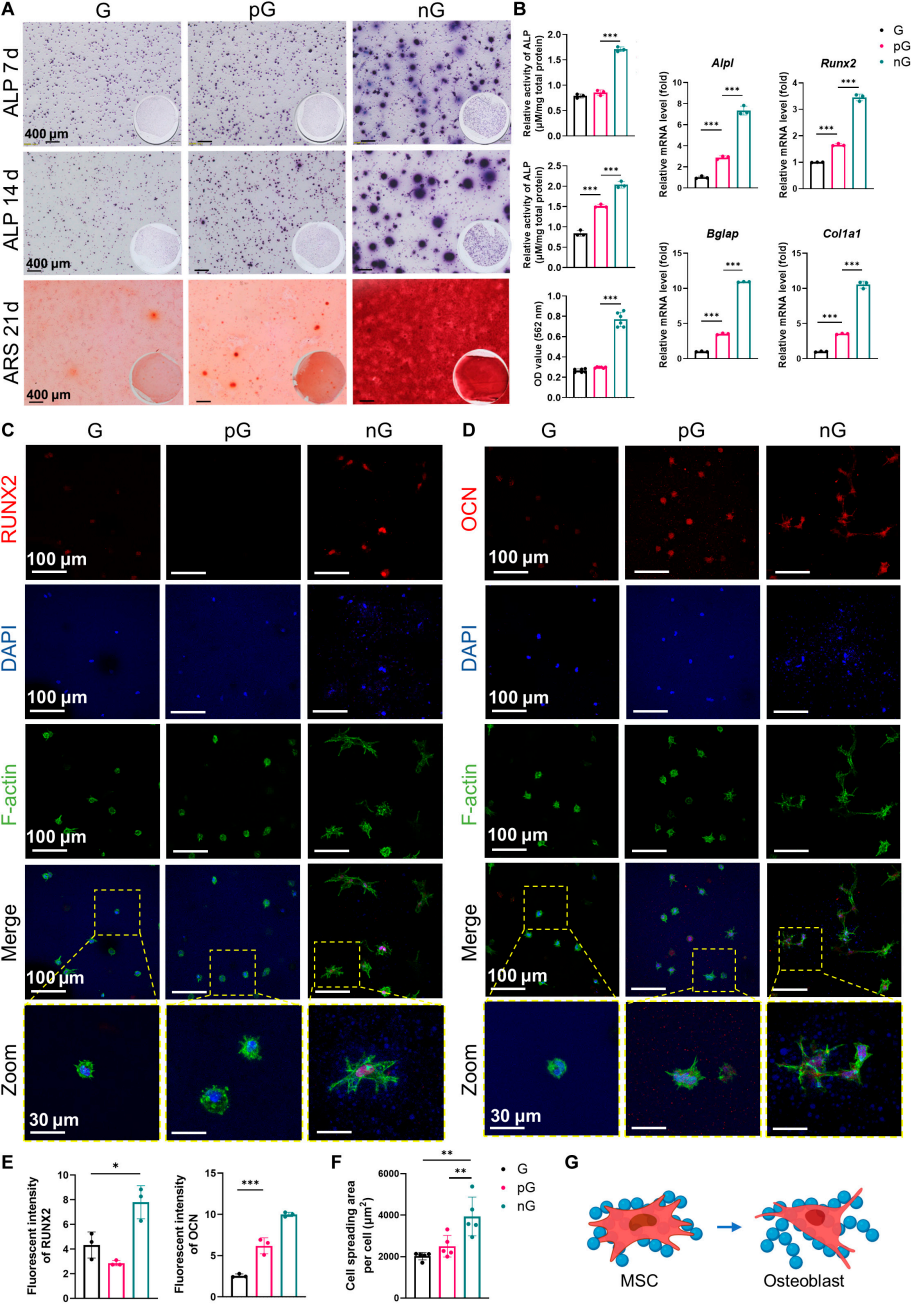
Enhanced osteogenic differentiation of rBMSCs by the cell-adaptable nG hydrogel. (A) Staining and quantitative analysis of ALP (*n* = 3) and ARS (*n* = 6). (B) Osteogenic gene expression of rBMSCs after a 2-week culture (*n* = 3). (C and D) Elevated expressions of RUNX2 and OCN in the nG hydrogel, respectively. (E) Fluorescent intensity of RUNX2 and OCN (*n* = 3). (F) Cell spreading area in hydrogels (*n* = 5). (G) rBMSCs exhibited morphology similar to osteoblasts after being cultured in the nG hydrogel (created with BioRender.com). *P < 0.05, **P < 0.01, and ***P < 0.005.

As previously noted, cells exhibited superior migration and spreading within the nG hydrogel, which is crucial for the viability and differentiation of rBMSCs. We further investigated the osteogenic potential of rBMSCs cultured in these hydrogels using 3D bioprinting. Notably, the nG hydrogel exhibited significantly enhanced ALP activity and conspicuous calcium nodule formation compared to the G and pG hydrogels (Fig. 5A). PCR analyses revealed substantial up-regulation of *Alpl*, *Runx2*, *Bglap*, and *Col1a1* in the nG hydrogel, with expression levels being 7.339-, 3.45-, 10.95-, and 10.53-fold higher than those in the G hydrogel at 14 d, respectively (Fig. 5B). Immunofluorescent staining corroborated these findings, showing significantly intensified expressions of RUNX2 and OCN in the nG hydrogel (Fig. 5C to G). Moreover, rBMSCs in the nG hydrogel displayed increased spreading area, dendrite, and filopodia, resembling the morphology typical of osteoblasts (Fig. 5F and G). The pG hydrogel showed limited promotion of osteogenic differentiation, due to pore walls that hindered optimal cell spreading and interactions.

### The cell-adaptable nG hydrogel promoted recruitment of MSCs in vivo

**Fig. 6.**
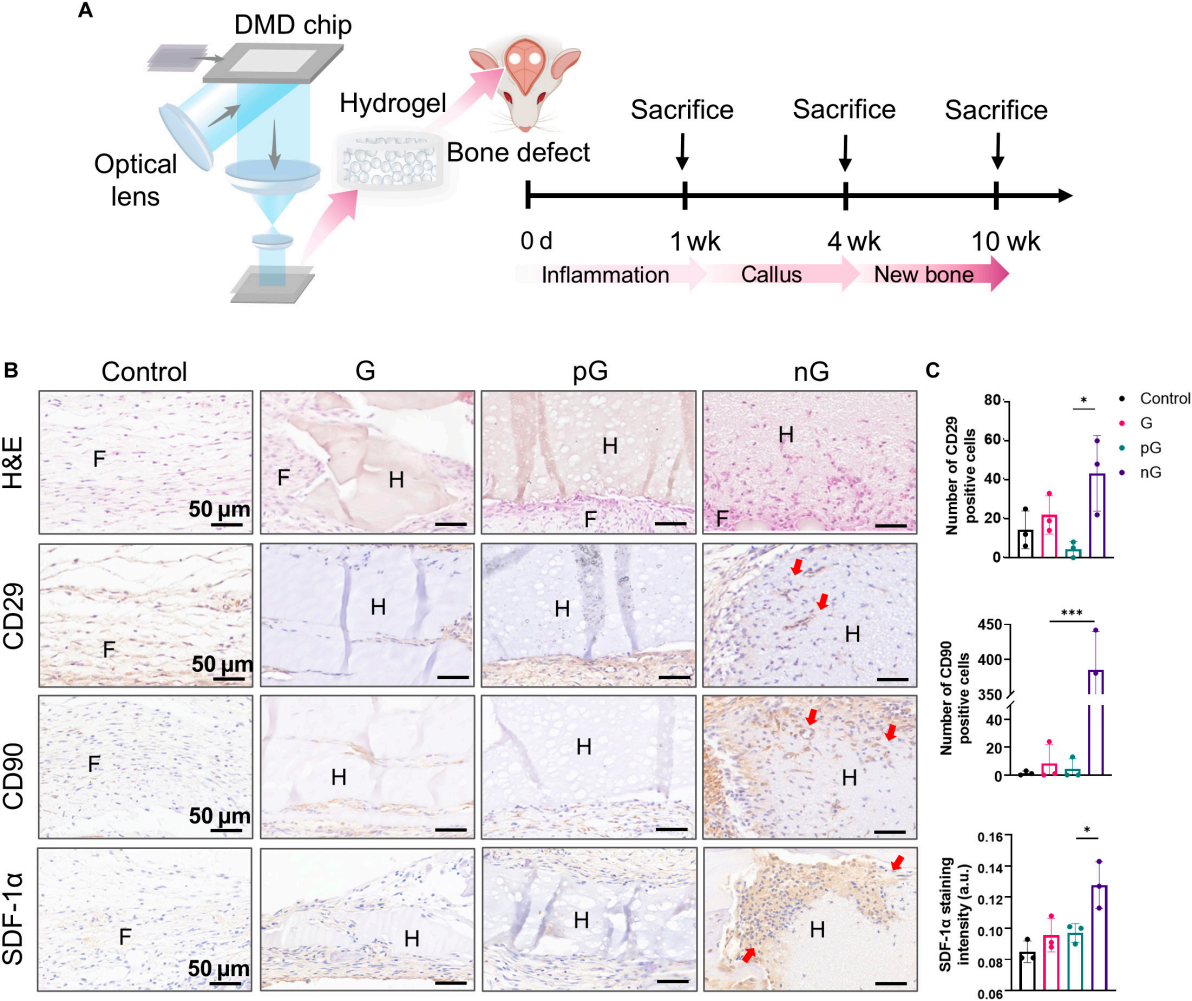
Recruitment of stem cells in calvarial bone defect area by the cell-adaptable nG hydrogel. (A) Schematic diagram of the animal study (created with BioRender.com). (B) H&E staining and IHC staining of CD29, CD90, and SDF-1α. Red arrows indicate positive expression. F, fibers; H, hydrogel. (C) Quantification of CD29^+^ and CD90^+^ cells, and expression of SDF-1α (*n* = 3). Statistical significance at *P < 0.05 and ***P < 0.005.

To investigate the effects of hydrogels on bone repair, we established critical-sized calvarial bone defects in rats, each with a diameter of 5 mm (Fig. 6A). Cell-free 3D-printed hydrogels were implanted into the defect areas. By day 7, H&E staining revealed that cells were recruited into the nG hydrogel, whereas they merely wrapped around the G and pG hydrogels (Fig. 6B). IHC staining targeting MSC markers showed an increase in CD29^+^ and CD90^+^ cells around and within the nG hydrogel. Furthermore, the pronounced expression of SDF-1α, a chemokine crucial for stem cell homing, was observed in the nG hydrogel (Fig. 6B and C). These findings suggest that the nG hydrogel is particularly effective at recruiting MSCs, which are likely to infiltrate into this hydrogel.

### The cell-adaptable nG hydrogel facilitated repair of critical-sized bone defects

**Fig. 7.**
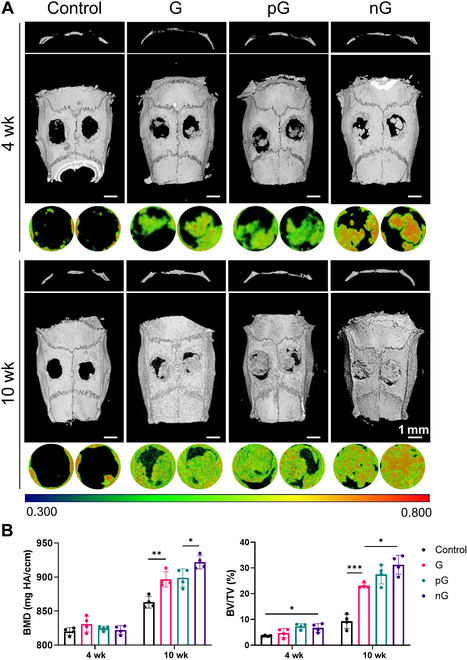
The μCT analysis of rat calvarial critical defects. (A) 3D reconstruction from μCT analysis. (B) Quantitative analysis of BMD and BV/TV (*n* = 4). Statistical significance at *P < 0.05, **P < 0.01, and ***P < 0.005.

**Fig. 8.**
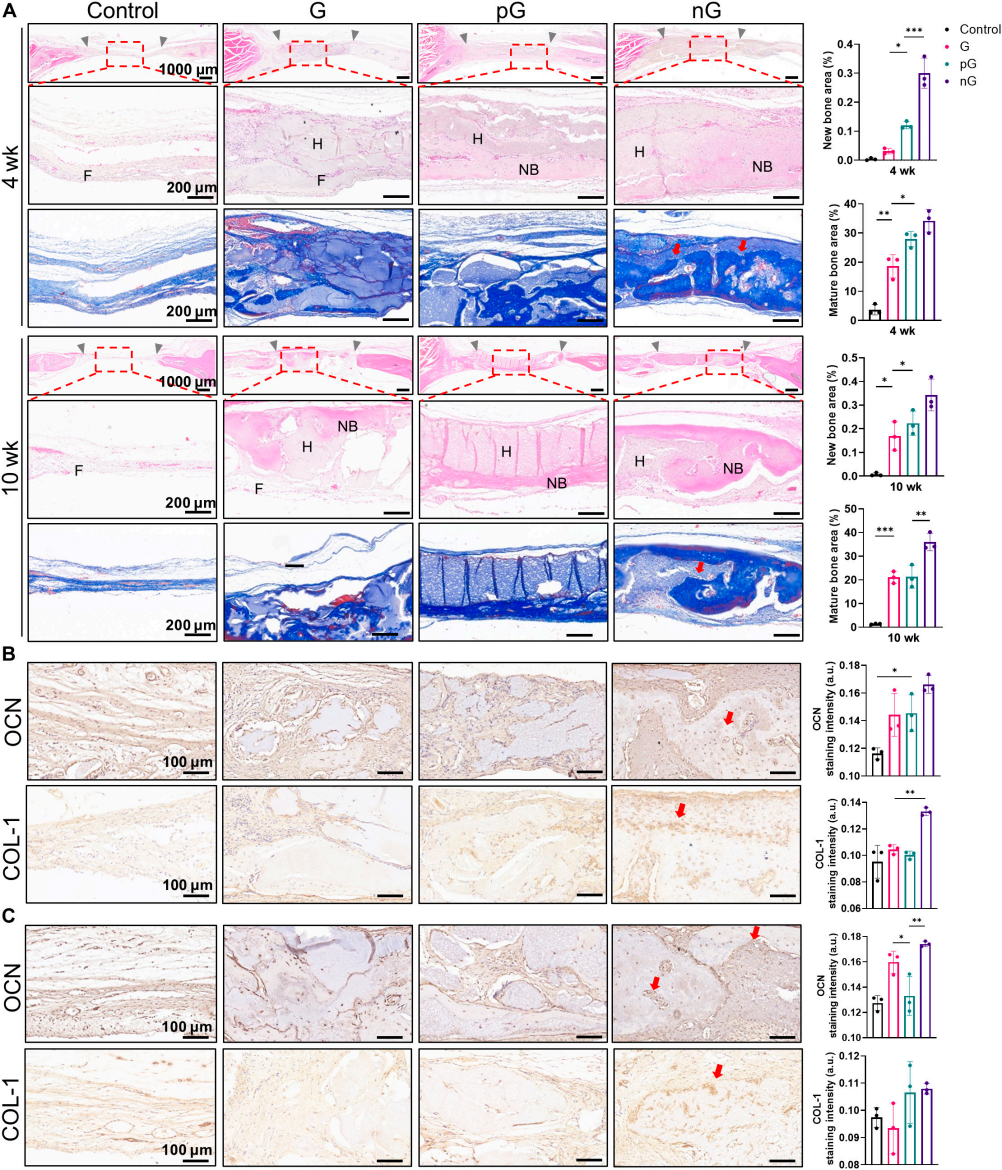
Histological and immunohistochemical staining of calvarial bone defects. (A) H&E and Masson staining at 4 and 10 weeks after surgery (*n* = 3). Gray arrows indicate the boundary of the defects. Red arrows indicate new bone deposition within the hydrogel. (B and C) IHC staining of OCN and COL-1 at 4 and 10 weeks after surgery, respectively (*n* = 3). Red arrows indicate positive expression around cells within the hydrogel. F, fibers; H, hydrogel; NB, new bone. Statistical significance at *P < 0.05, **P < 0.01, and ***P < 0.005.

The effects of hydrogels on bone regeneration in rat calvarial bone defects were evaluated in vivo using μCT at 4 and 10 weeks after surgery. In comparison to the control group, the G hydrogel displayed a modest degree of bone repair. Both the pG and nG hydrogel promoted bone regeneration, with the nG hydrogel showing nearly complete coverage of the defect area with new bone at 10 weeks (Fig. 7A). The nG hydrogel also yielded significantly higher values of BMD (Fig. 7B). Furthermore, H&E and Masson staining revealed that collagen fibers grew into the G and pG hydrogels, with new bone formation primarily occurring around these fibers. Notably, new bone deposition in the nG hydrogel was observed not only around but also within the material, likely due to the cellular distribution throughout the hydrogel (Fig. 8A). IHC staining further indicated stronger expression of OCN and COL-1 in the nG hydrogel, particularly around the cells embedded in the hydrogel (Fig. 8B and C). Thus, the cell-adaptable nG hydrogel facilitated endogenous bone formation in critical-sized bone defects.

### The nG hydrogel up-regulated the expressions of factors for mechanotransduction

**Fig. 9.**
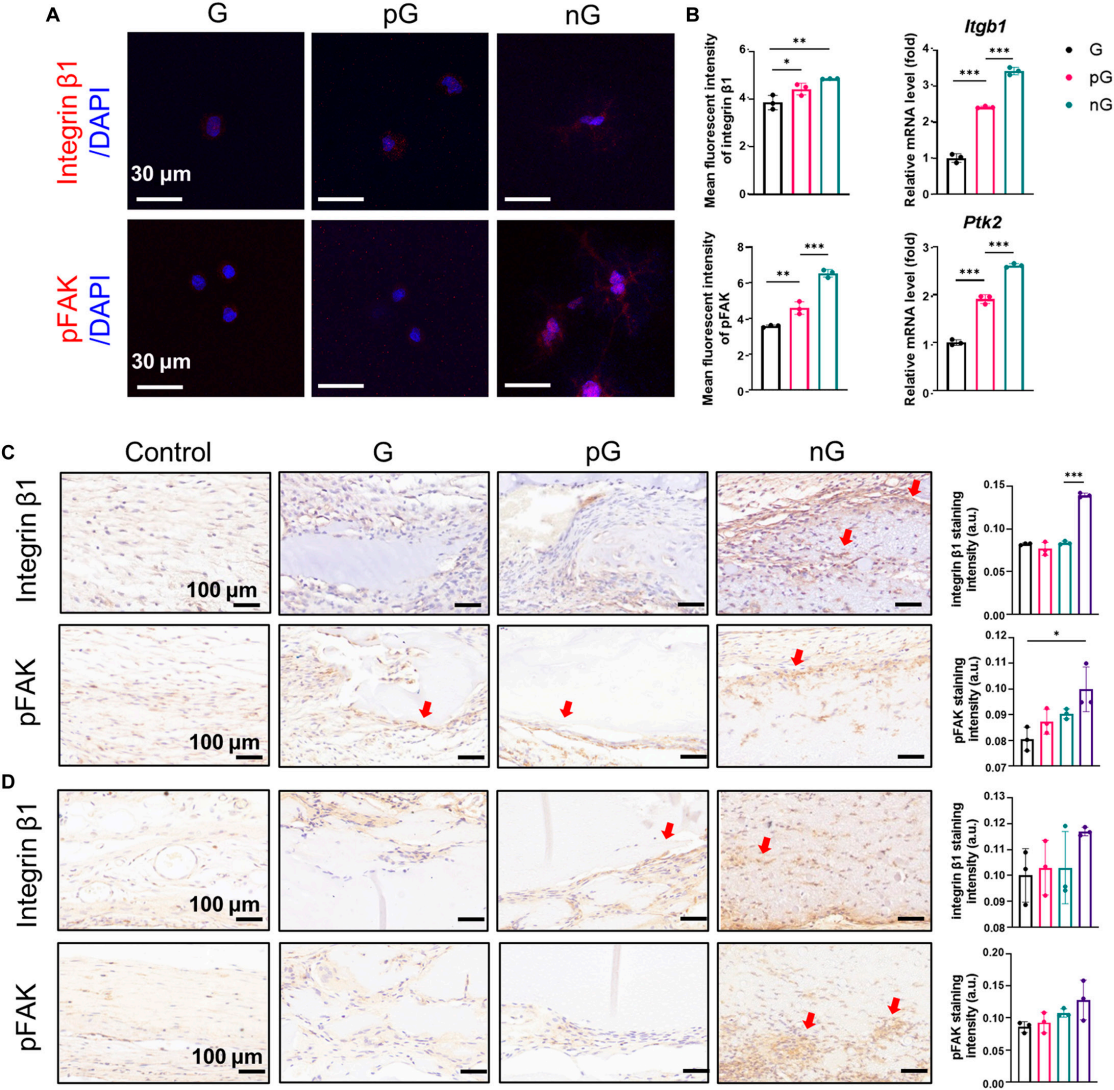
Up-regulation of key factors for ECM–intracellular mechanotransduction by the cell-adaptable nG hydrogel. (A) Immunofluorescent staining of integrin β1 and pFAK in cells cultured in hydrogels on day 3. (B) Gene expression analysis of *Itgb1* and *Ptk2* in cells cultured in hydrogels on day 3 (*n* = 3). (C and D) IHC staining of integrin β1 and pFAK in the calvarial bone defect area at 7 d and 4 weeks after surgery (*n* = 3). Red arrows indicate positive expression. Statistical significance at ***P < 0.005.

In this study, ICC staining showed enhanced expression of integrin β1 and phosphorylated FAK (pFAK) in rBMSCs cultured in the nG hydrogel through 3D bioprinting after 3 d (Fig. 9A). Similarly, both protein and gene expression of these factors were up-regulated, as demonstrated by WB and PCR analyses in vitro (Fig. 9B and Fig. S6). Moreover, IHC staining of rat calvarial bone defects revealed extensive expression of integrin β1 and pFAK in the nG hydrogel in vivo at 7 d and 4 weeks after surgery (Fig. 9C). By 10 weeks after surgery, when the nG hydrogel had largely been converted to new bone, the expression level showed no significant difference among all groups (Fig. S7). These findings suggested that the specific nanocolloidal structure of the nG hydrogel may enhance migration, spreading, and osteogenic differentiation of MSCs, at least partially through the activation of integrin/FAK signaling pathway.

## Discussion

Critical-sized bone defects, which cannot be spontaneously repaired by the body, pose great challenges in clinical treatment [20]. Biomaterials capable of filling bone defects while recruiting endogenous stem cells and promoting their osteogenic differentiation offer a promising therapeutic strategy. Once scaffolds are implanted at bone defect sites, new bone gradually deposits from the outer edges toward the center as the material degrades, facilitating bone repair. However, in critical or large-sized bone defects, the center remains distant from blood supply and nutrients, impeding cell growth and differentiation, slowing the bone repair process [21]. Therefore, guiding endogenous stem cells to infiltrate scaffolds, followed by their proliferation and osteogenic differentiation, could lead to more efficient bone regeneration. Stem cell functions are influenced by factors such as ECM, bioactive signals (cytokines, hormones, etc.), and biomechanical cues [22]. Although extensive studies have explored recruiting stem cells and directing their differentiation through exogenous factors, this approach is limited by high costs and associated risks. Consequently, there is a growing trend toward utilizing the physical properties of materials to flexibly regulate stem cell functions. Inspired by the porous, nanoscale, and dynamic microstructure of natural ECM, this study developed an nG hydrogel with customizable shapes. The hydrogel was fabricated using a bioink composed of GelMA and F68, and synthesized into customizable macroscopic shapes that simultaneously incorporated nanospheres through DLP-based 3D printing. This method is more efficient and environmentally friendly than traditional methods, which typically require high temperatures, organic solvents, or high-speed dispersion to synthesize nanoparticles, followed by their assembly into colloidal networks. Compared to other 3D printing techniques, the DLP-based 3D printing offers superior performance in fabrication speed, printing accuracy, structural complexity, and integrity [23].

Generally, the porous framework of ECM plays a pivotal role in supporting cellular proliferation, migration, and nutrient and metabolite exchange, thereby expediting bone regeneration [24]. The distinct microscopic structures of the 3 hydrogels arise from their different formation mechanisms. Compared to the G hydrogel, the pG hydrogel had pores generated by immiscibility between GelMA and PEO solution [19]. However, the pores in the pG hydrogel were isolated with walls between them [25], resulting in suboptimal pore connectivity compared to the nG hydrogel. The nG hydrogel was composed of interconnected GelMA nanospheres formed through phase separation between GelMA and F68. GelMA, known for its biocompatibility, retains gelatin’s key components, such as the arginine–glycine–aspartic acid (RGD) peptide sequence for cell adhesion and matrix metalloproteinase (MMP)-sensitive sites for enzyme-mediated degradation [26,27]. F68, a Food and Drug Administration (FDA)-approved surfactant, has a centrally hydrophobic PEO chain flanked by 2 hydrophilic polypropylene oxide (PPO) chains [28]. When GelMA and F68 are thoroughly mixed, GelMA nanospheres self-assemble and gel through photo-crosslinking. These nanospheres created interconnected pores and increased the specific surface area, significantly enhancing cell proliferation, spreading, and migration. Moreover, unlike dispersed nanoparticles, these interconnected nanospheres do not raise potential toxicity concerns, such as endocytosis, in in vivo applications [29]. H&E staining revealed that the nG hydrogel showed no significant inflammatory response after subcutaneous implantation, degraded at 28 d partially, and completely degraded at 56 d. In bone repair process, the first 2 weeks primarily involve the inflammatory phase, along with the formation of new blood vessels and fibrous tissue. From 2 weeks to 6 months after injury, new bone begins to form. Therefore, biomaterials must gradually degrade during this period to provide space for cell infiltration and tissue ingrowth [2]. Thus, the nG hydrogel exhibited an ideal degradation profile that supports bone regeneration.

In addition to porous structure, the nanostructures in scaffolds can enhance hydrophilicity and the adsorption of organic molecules and inorganic ions, such as proteins and calcium ions, attributes that synergistically promote cell adhesion, migration, and differentiation [3]. Research has shown that MSCs develop more filopodia on nanostructured substrates, influencing stem cell fate [30]. Nanostructures that mimic natural ECM and activate cellular receptors at the nanoscale serve as effective inducers of cell differentiation [31]. Various nanostructures, including nanopatterned patches [32], nano-furrow [33], and tubular nanomaterials [34], have been shown to promote osteogenic differentiation [35]. Similarly, the nanospheres in the nG hydrogel significantly enhanced the adhesion and osteogenic differentiation of rBMSCs compared to the G and pG hydrogels [36].

The natural ECM not only contains robust fibrous components that preserve the static morphology of tissues and the micro-/nanostructure but also includes polysaccharides and proteoglycans interspersed among the fibers. These elements support a dynamic microscopic structure that responds to cellular changes [37]. In this study, GelMA nanospheres were semi-crosslinked to form the nG hydrogel. We first confirmed its dynamic structure through frequency sweep analysis. The observed changes in cell migration patterns and morphology, as well as the changes of pores and nanospheres in the nG hydrogel, further validated its cell-adaptable dynamic characteristics. Designing cell-adaptable structures facilitates diverse cell motility, enabling cell growth, migration, and alterations in cellular morphology [38]. Studies have found that such cell-adaptable structures enhance cell–cell and cell–ECM interactions, which influence cellular energy metabolism and gene expression through mechanotransduction. This process ultimately modulates cell behaviors, including osteogenic differentiation [8,39].

Fundamentally, cells interact with the ECM through surface receptors, with integrin serving as a pivotal mediator in facilitating mechanical signal transduction [40]. Upon activation, FAK and other adhesion-related proteins, including vinculin, talin, and paxillin, bind to integrin. This interaction generates contractile forces transmitted to the cytoskeleton and nucleus, modulating cellular functions. Among these proteins, FAK plays a critical role in integrin-mediated cellular processes, primarily through its phosphorylation, which regulates key activities such as cell adhesion, migration, and differentiation. Studies have shown that reduced pFAK expression impairs cell motility. Silencing FAK markedly decreases the expression of ALP and RUNX2 in MSCs, and the expression of OPN and COL-1 in osteoblasts [32]. Increased expression levels of integrin and pFAK, as well as the osteogenic-related factors have been observed in stem cells on scaffolds with micro-/nanostructures [41,42], which is consistent with our findings. Additionally, the cell-adaptable dynamic microstructure can enhance the mechano-sensing process of encapsulated MSCs, thereby further promoting osteogenic differentiation [43]. In this study, the unique static and dynamic microscopic features of the nG hydrogel, including its interconnected nanospheres and cell-adaptable characteristics, triggered the expression of osteogenic-related proteins at least partially under the activation of integrin β1/FAK mechanotransduction signaling pathway.

Moreover, the biomechanical cues influence various cellular interactions through integrin/FAK signaling pathway. As is known, macrophages play a critical role in bone defect repair. Studies suggest that nano-roughness can induce macrophage M2 polarization through integrins and focal adhesion molecules for enhanced bone regeneration. Additionally, MSC-mediated suppression of inflammatory cytokine production by immune cells may be regulated via the FAK–COX2 signaling pathway, fostering a favorable osteo-immune microenvironment [5]. Besides macrophages, mechanotransduction through the FAK–VEGF signaling pathway impacts endothelial cell functions and angiogenesis, further contributing to new bone formation [44,45]. Overall, the nG hydrogel appears to exert its effects through complex interactions involving multiple cell types [20]. This study has only revealed a small part of the biomechanistic scope, highlighting the need for further elucidation in the future.

In conclusion, while the mechanical performance of the nG hydrogel does not match that of natural bone [46], its porous, cell-adaptable nanocolloidal structure effectively induced endogenous bone formation both around and within the hydrogel. This was achieved by enhancing the recruitment and infiltration of endogenous MSCs and promoting their osteogenic differentiation. The strong osteoinductive potential of the nG hydrogel is likely attributed to the activation of the integrin β1/FAK mechanotransduction signaling pathway, triggered by biomechanic stimuli from the nanostructure and dynamic nanocolloidal network. Thus, the 3D-printed cell-adaptable nG hydrogel demonstrates multifunctional osteoinductive capabilities, acting as a “bio-sponge” to facilitate endogenous bone formation in critical-sized bone defects, particularly in non-load-bearing regions. Future efforts will focus on integrating the nG hydrogel with high-strength materials, such as guided bone regeneration membranes and titanium meshes, which lack sufficient osteoinductive capability [47]. This approach aims to provide both self-supporting properties and enhanced bone regeneration, expanding the clinical applications of the nG hydrogel.

## Ethical Approval

This study was approved by the Animal Care and Ethics Committee of West China School of Stomatology, Sichuan University (WCHSIRB-D-2022-583). All animal experiments complied with the Animal Research: Reporting of In Vivo Experiments (ARRIVE) guidelines.

## Data Availability

The datasets used and analyzed during the current study are available from the corresponding author on reasonable request.
