# Supplementary material for: Three-Dimensional Printed Cell-Adaptable Nanocolloidal Hydrogel Induces Endogenous Osteogenesis for Bone Repair
Source: Biomater Res. 2025 Feb 14;29:0146. doi: 10.34133/bmr.0146 (PMC11825971; doi:10.34133/bmr.0146)
Supplement: Supplementary 1 — Figs. S1 to S7 Table S1 [file bmr.0146.f1.docx]

**Supporting Information**


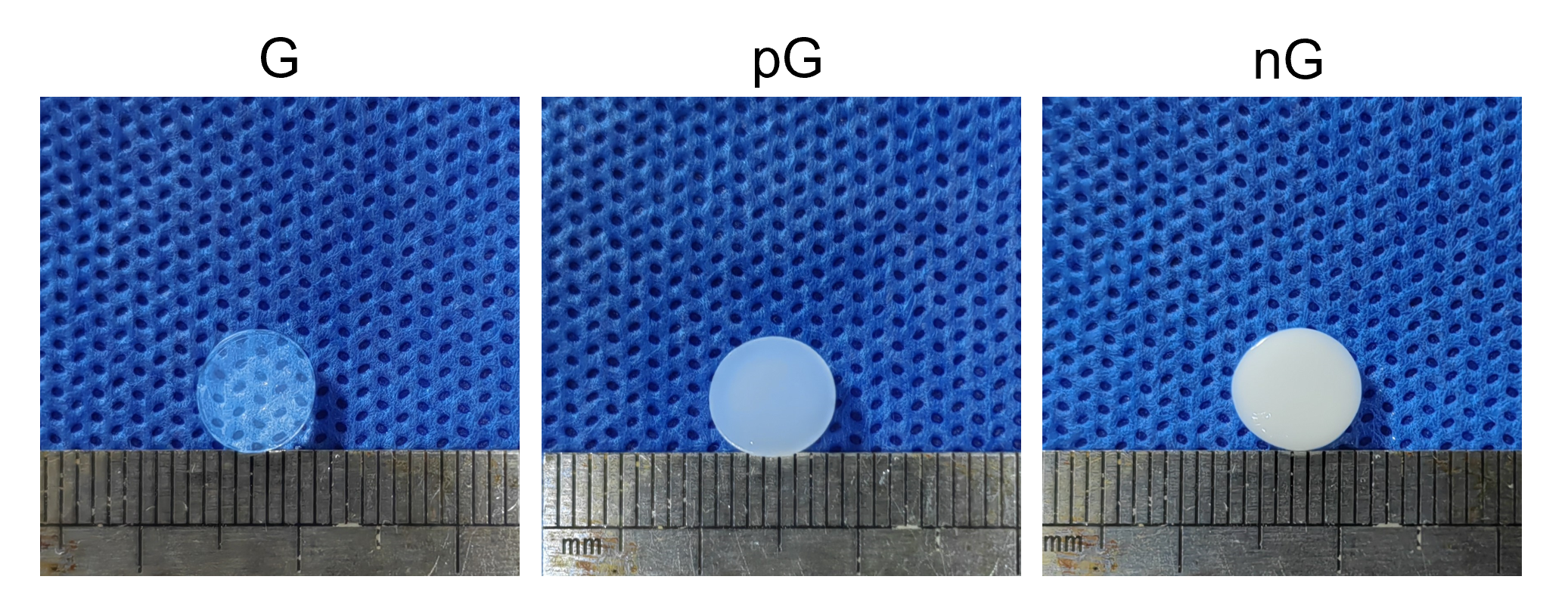


**Fig. S1.** Appearance of hydrogels prior to subcutaneous implantation.


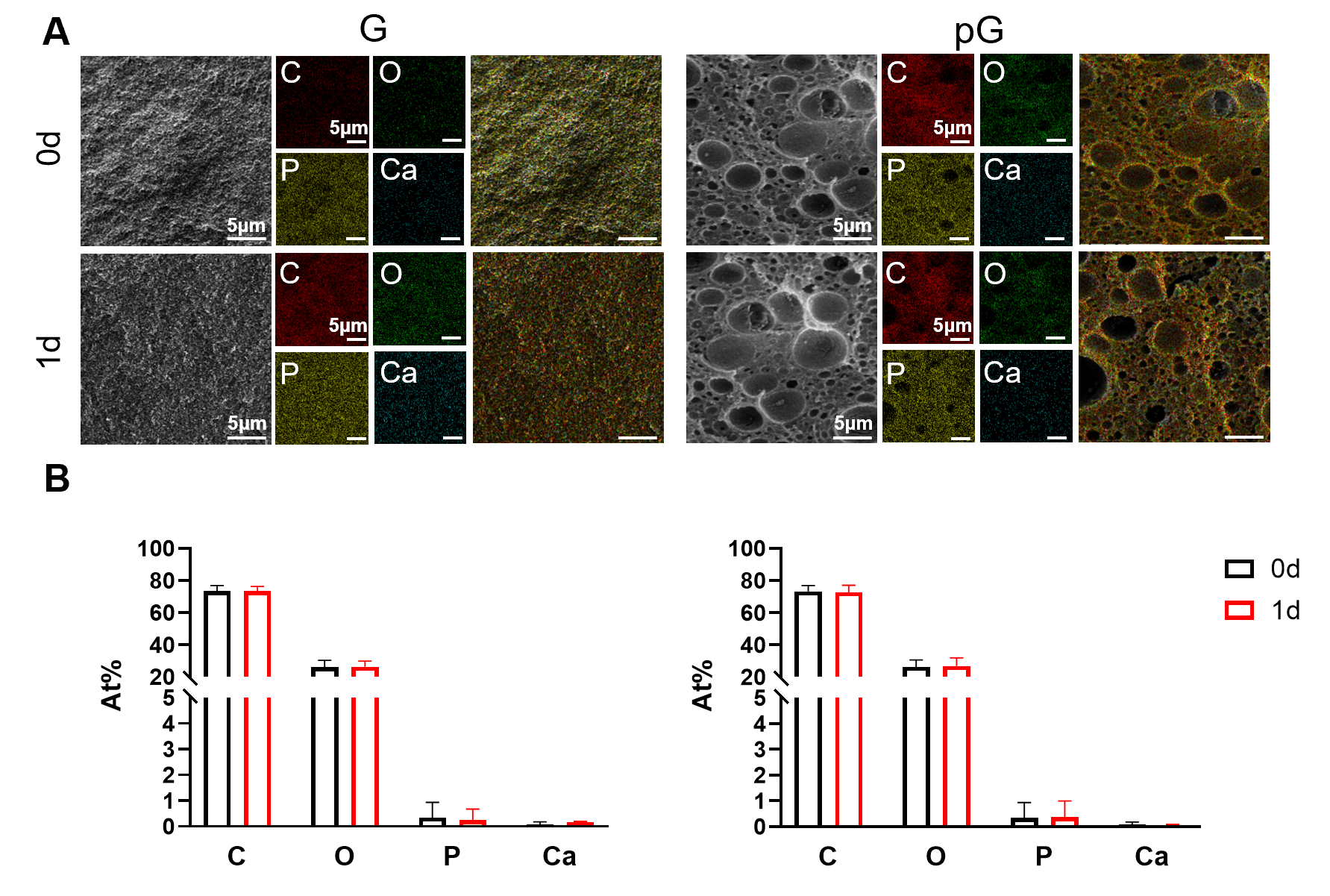


**Fig. S2.** EDS analysis of the G and pG hydrogel before and after mineralized in 1.5×SBF for 1 day. (A) elemental mapping images. (B) Atoms percentages of carbon (C), oxygen (O), phosphorus (P), and calcium (Ca).


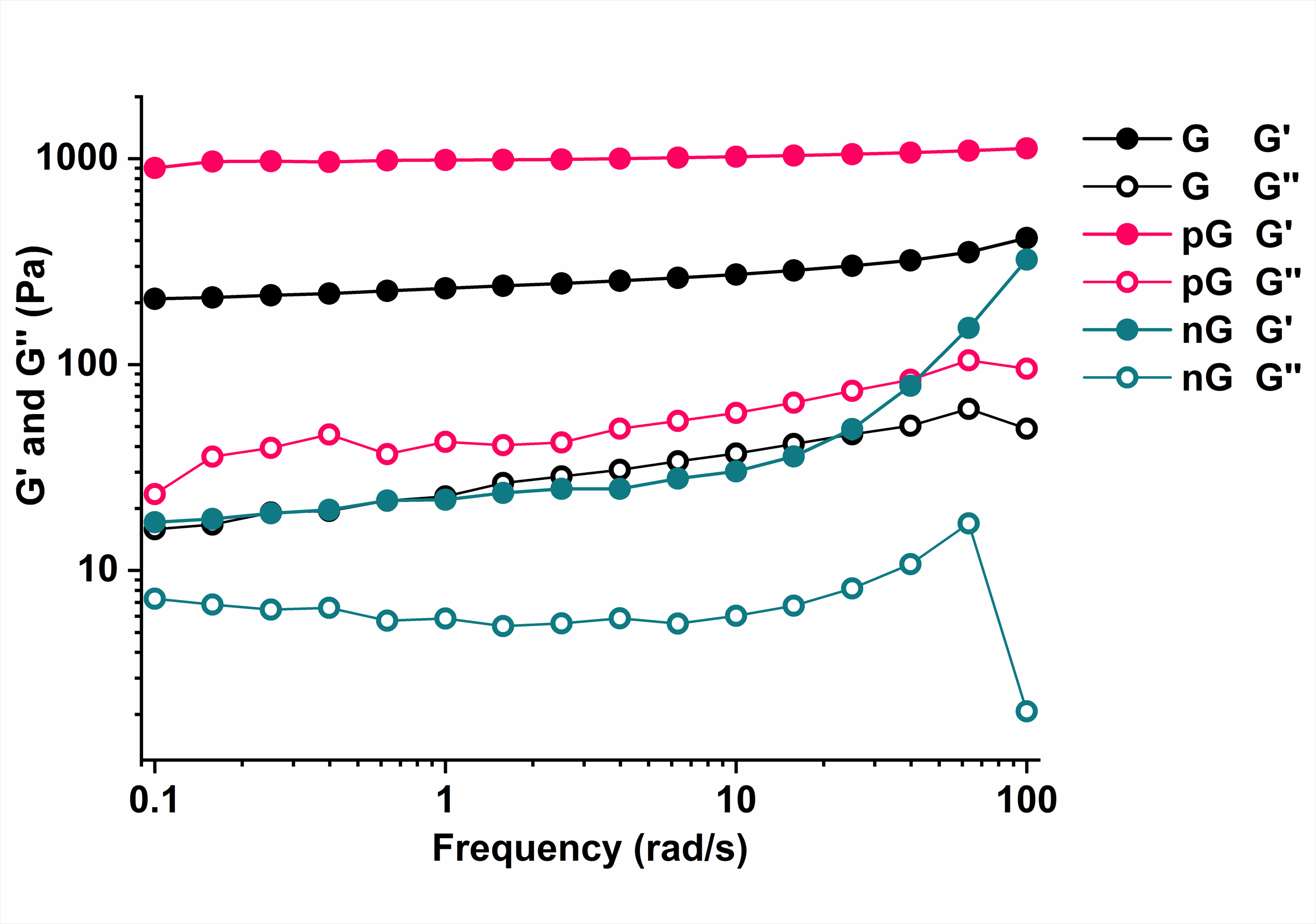


**Fig. S3.** The storage (G′) and loss (G″) moduli of hydrogels according to frequency sweep analysis in rheological tests.


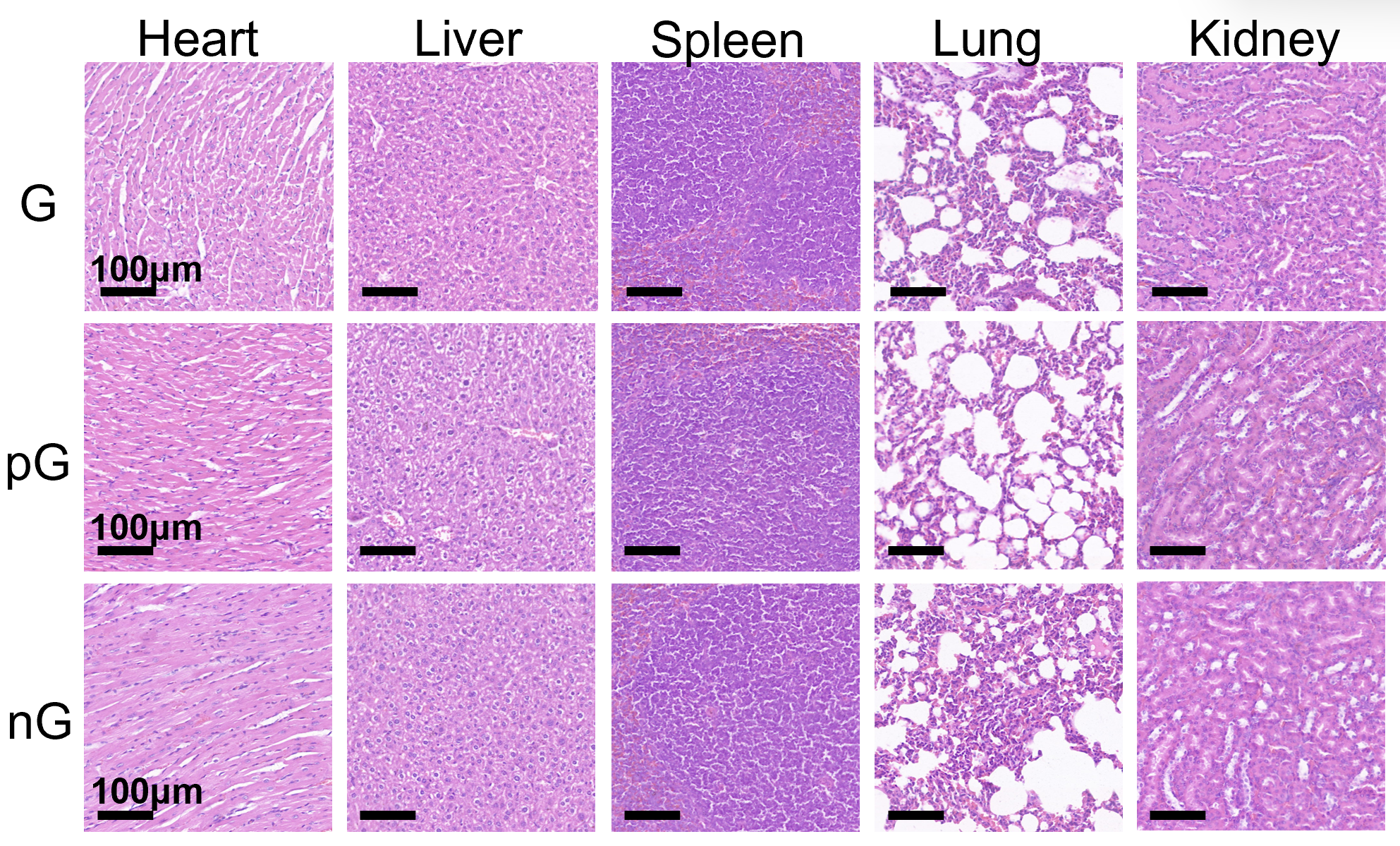


**Fig. S4.** The HE staining of vital organs at 1 week after hydrogel implantation.


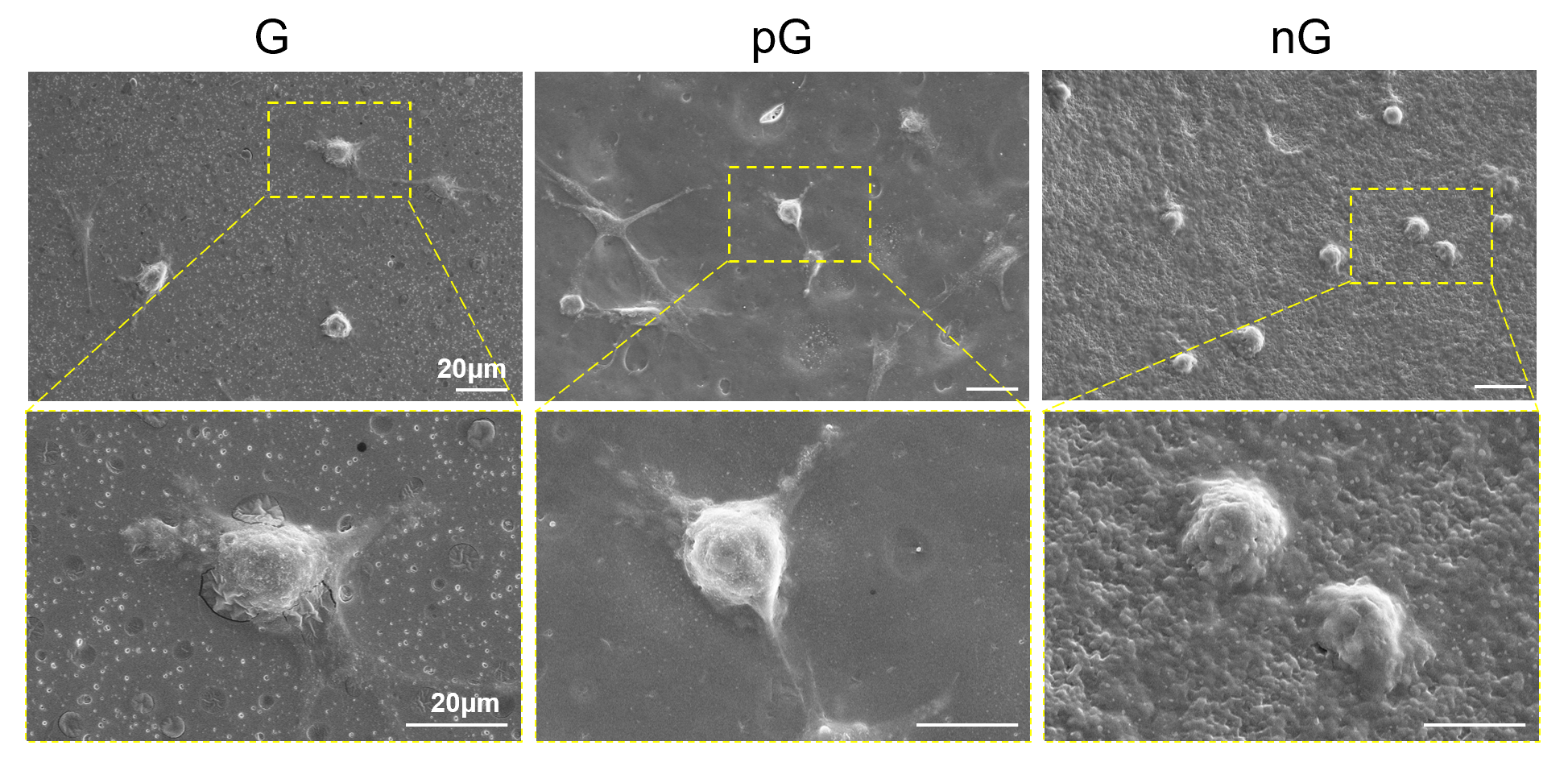


**Fig. S5.** SEM images of rBMSCs seeded on hydrogel surfaces within the first 3 hours.


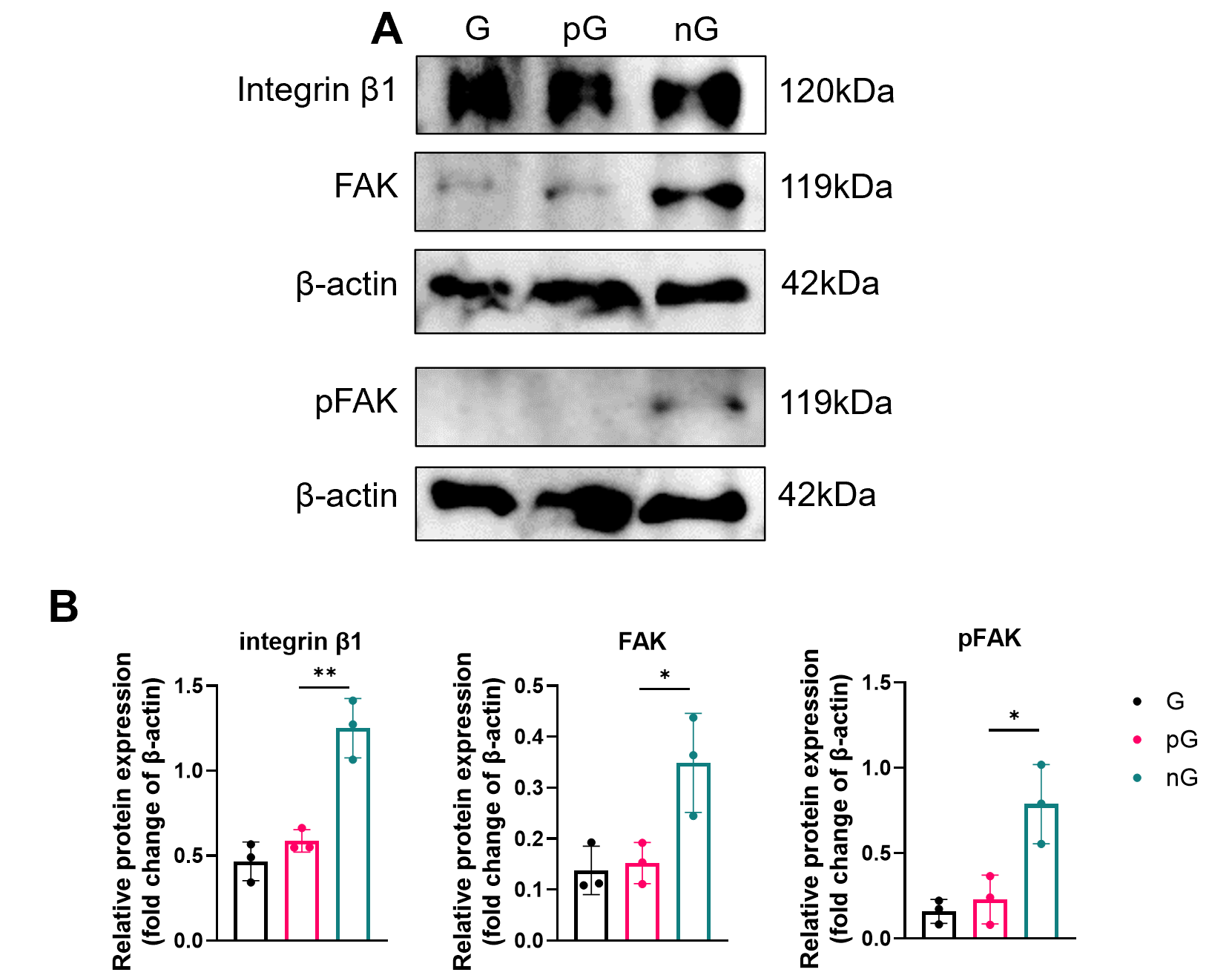


**Fig. S6.** WB analysis of integrin/FAK pathway of cells cultured within hydrogels on day 3. (A) WB images. (B) The quantitative analysis of integrin/FAK pathway. Statistical significance at **P* < 0.05 and ***P* < 0.01.


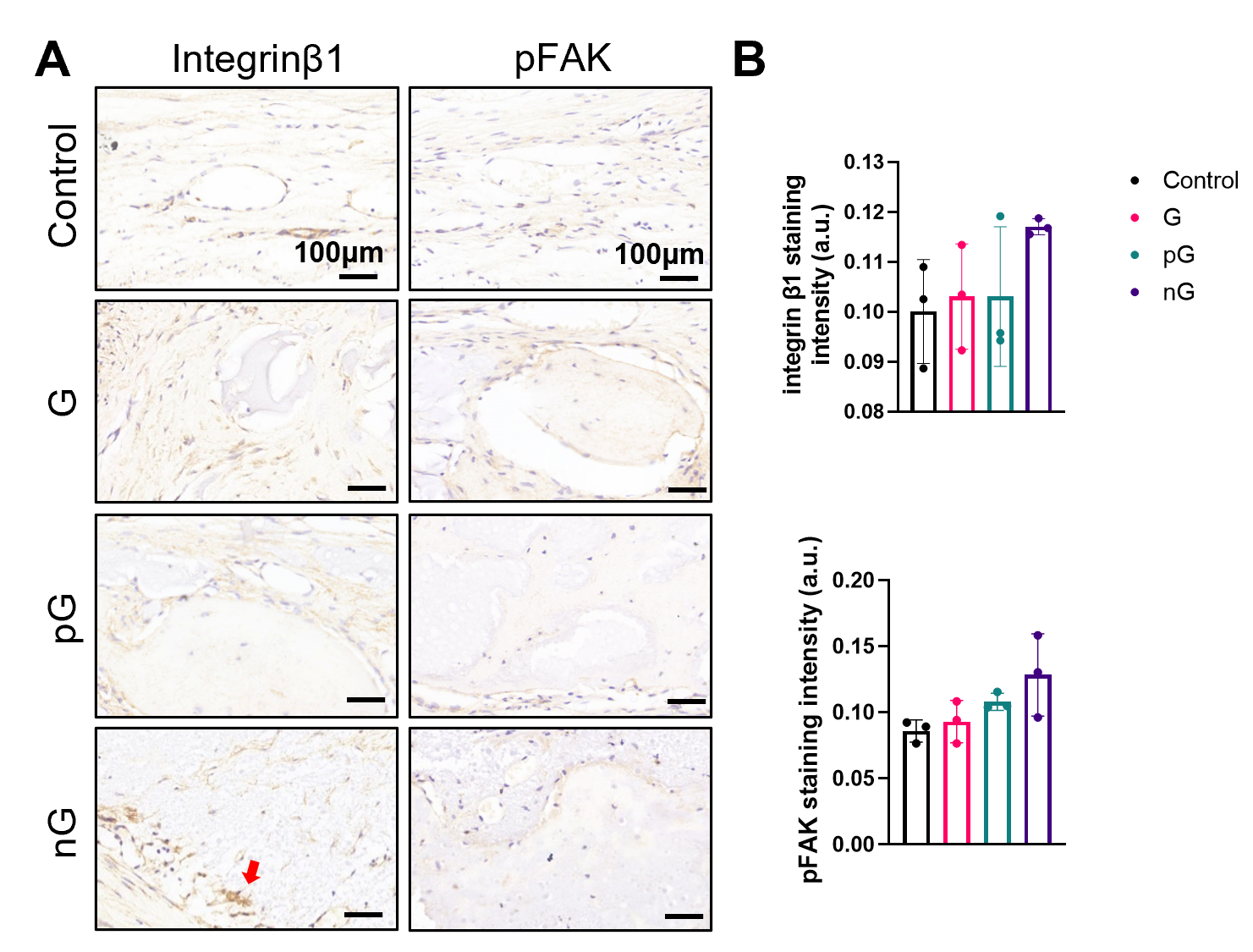


**Fig. S7.** IHC staining of integrin β1 and pFAK of calvarial bone defect area at 10-week post-surgery. (A) The IHC staining. (b) The quantitative analysis (*n* = 3). Statistical significance at **P* < 0.05, ***P* < 0.01, and ****P* < 0.005.

**Table S1.** Primer sequences.

| **Gene** | **Primer sequences (5’-3’)** |
| --- | --- |
| *Gadph* | Forward: GGGTGTGAACCACGAGAAAT |
|  | Reverse: ACTGTGGTCATGAGCCCTTC |
| *Alpl* | Forward: CACGGCGTCCATGAGCAGAAC |
|  | Reverse: CAGGCACAGTGGTCAAGGTTGG |
| *Runx2* | Forward: CTCTTCCCAAAGCCAGAGCG |
|  | Reverse: ACCATCCTGGAAGGAGACCG |
| *Bglap* | Forward: AGACTCCGGCGCTACCTCAAC |
|  | Reverse: GGCGTCCTGGAAGCCAATGTG |
| *Col1a1* | Forward: TGTTGGTCCTGCTGGCAAGAATG |
|  | Reverse: GTCACCTTGTTCGCCTGTCTCAC |
| *Itgb1* | Forward: CTGCGATAGGTCCAACGGCTTAATC |
|  | Reverse: GGATAGCATTCACAGACACGACACC |
| *Ptk2* | Forward: ACCTAAGCAACTTGTCCAGCATCAG |
|  | Reverse: GGATCGGTCAAGGTTGGCAGTG |
